# Chemical Profile and Biological Activities of Essential Oil from *Piper arboreum* for Development and Improvement of Mouthwash

**DOI:** 10.3390/molecules27196408

**Published:** 2022-09-28

**Authors:** Edinardo Fagner Ferreira Matias, Ana Paula Dantas Pereira, Ana Valéria de Oliveira Braz, Mariana Carvalho Rodrigues, Jussara de Lima Silva, Philippe Alencar Araujo Maia, Sarah Castro dos Santos, Ricardo Andrade Rebelo, Ieda Maria Begnini, Luiz Everson da Silva, Wanderlei do Amaral, Grażyna Kowalska, Rafał Rowiński, Joanna Hawlena, Radosław Kowalski, Henrique Douglas Melo Coutinho, Vilson Rocha Cortez Teles de Alencar

**Affiliations:** 1 Department of Biomedicine, University Center Dr. Leão Sampaio, Juazeiro do Norte 63040-405, CE, Brazil; 2 Dentistry Course, CECAPE College, Juazeiro do Norte 63024-015, CE, Brazil; 3 Department of Chemistry, Regional University of Blumenau, Rua Antônio da Veiga, 140, Blumenau 89030-903, SC, Brazil; 4 Post-Graduate Programme in Sustainable Territorial Development, Paraná Federal University, Matinhos 83260-000, PR, Brazil; 5 Department of Tourism and Recreation, University of Life Sciences in Lublin, 15 Akademicka Street, 20-950 Lublin, Poland; 6 Department of Analysis and Evaluation of Food Quality, University of Life Sciences in Lublin, 8 Skromna Street, 20-704 Lublin, Poland; 7 Department of Biological Chemistry, Regional University of Cariri—URCA, Crato 63105-000, CE, Brazil

**Keywords:** antibacterial activity, bacterial infection, resistance modulation, oral health, oral hygiene

## Abstract

Studies show that more consumers are using natural health products in the modern world. We have noticed a growing demand in markets and the professional community for mouthwashes that contain natural compounds. The objective of this study was to assess the chemical characterization and microbiological potential of the essential oil *Piper arboreum* (EOPa) to provide data to enable the development of a low-cost mouthwash. The evaluation of the antibacterial and bacterial resistance modulating activity was performed by the microdilution method to determine the minimum inhibitory concentration. The chemical components were characterized by gas chromatography coupled with mass spectrometry, which identified 20 chemical constituents, with caryophyllene oxide being one of the major compounds. The EOPa showed an MIC ≥ 1024 µg/mL for all bacterial strains used in the tests. When evaluating the modulating activity of EOPa combined with chlorhexidine, mouthwash and antibiotics against the bacterial resistance, the oil limited synergistic activity between the MIC of the products tested in combination (37% to 87.5%). Therefore, we recommend expanding the tests with greater variation in the EOPa concentration and the products used, as well as toxicity assessments and in vivo testing, with the purpose of the development of a possible low-cost mouthwash base that is accessible to the most vulnerable populations.

## 1. Introduction

Several scientific studies have confirmed the biological activities of essential oils, such as antidiabetic action [1], antioxidant [2], anxiolytic [3], sedative [4], anti-inflammatory [5], hepatoprotective [6], antitumor [7], gastroprotective [8], and hypolipidemic [9]. The antibacterial and antibiotic resistance modulating activities using natural products of plant origin have been repeated in studies on several parts of plants: leaf extract [10], leaf oil [11], extract from leaves and stem [12], oil from aerial parts [13] and oil from leaves, stem and roots [14].

Essential oils are complex mixtures extracted from plants that have several biological properties related to their survival and defense [15], however, the chemical constitution of these products varies according to the plant’s genotype [16], geographic origin [17], conditions, environment, season in the year [18], extraction method and conservation [19].

The Piperaceae family comprises 1100 plant species distributed in 12 genera, which includes the genus Piper with outstanding ethnobotanical and ethnopharmacological relevance is widely distributed in subtropical regions and known for its aromatic herbs. Popularly known as “pau-de-angola”, “jaborandi”, and “chili pepper”, the *Piper arboreum* species is related to several biological activities such as antifungal, trypanocidal, antibacterial and antioxidant [20,21]. The literature shows that the compounds bicyclogermacrene, E-caryophyllene, caryophyllene oxide and spathulenol are the most commonly found in the *Piper arboreum* species and can be extracted from dry leaves, fresh leaves and the stem of the plant [22].

The oral microbiota is composed of a wide variety of microorganisms, including several species of bacteria, fungi, viruses and protozoa, which can often act as beneficial agents, preventing the colonization of the oral cavities by pathogenic microorganisms [23].

*Staphylococcus aureus* is a Gram-positive bacterium, that is a constituent of the natural microbiota of the skin and nasal mucosa and is considered an opportunistic pathogen that is frequently associated with infections acquired in community and hospital environments [24,25,26,27]. *Streptococcus mutans*, one of the several etiological factors of tooth decay, is capable of colonizing the oral cavity and forming a bacterial biofilm. Additional properties that allow *S. mutans* to colonize the oral cavity include the ability to survive in an acidic environment and the specific interaction with other microorganisms that colonize this ecosystem [28,29,30,31]. 

Mechanical disruption of the biofilm with brushing and interdental brushing is currently the best prevention method to prevent and reduce gingival inflammation. This mechanical action is insufficient without the use of chemical products such as dentifrices [31,32].

Chemical agents such as triclosan, sodium lauryl sulfate (SLS) and propylparaben, as well as allergens such as methylisothiazolinone, methylchloroisothiazolinone and chlorhexidine are commonly added to toothpastes to enhance their antibacterial action. These products may pose a risk to human health. Some of these substances have undesirable side effects, such as changes in taste and stains on teeth, and doubts persist about the harmful impacts on endocrine function, notably on fertility. Some manufacturers have moved away from SLS, chlorhexidine and triclosan and have introduced other less irritating surfactants such as nonionic polyethylene glycol ethers of stearic acid [33,34,35,36,37]. 

The antimicrobial agents present in toothpaste cannot effectively penetrate areas of difficult access in the oral cavity, resulting in the accumulation of bacteria that inhabit the biofilm in the interdental spaces [38,39]. In this sense, mouthwashes for daily use are a complement to brushing to improve oral health [40,41,42].

Given the arguments presented, the research aimed to assess the chemical characterization and microbiological potential of the essential oil of *Piper arboreum* and provide data to enable the development of a low-cost mouthwash formulation aimed at vulnerable communities.

## 2. Results

After GC-FID and GC-MS analysis of the essential oil sample of *Piper arboreum* (EOPa), 20 chemical constituents were identified corresponding to 67.53% of the total composition of the sample, as shown in Table 1. In this chemical analysis, caryophyllene oxide (No. 18) was a major compound (30.50%), followed by myristicin (No. 14), spathulenol (No. 17), E-caryophyllene (No. 5) and humulene Epoxide II (No. 19), with 7%, 6.20%, 5.10% and 5.10%, respectively.

When evaluating the antibacterial potential of the essential oil of *Piper arboreum* (EOPa), with the determination of the minimum inhibitory concentration (MIC), the EOPa presented MIC ≥ 1024 µg/mL for all bacterial strains used in the tests.

**Figure 1:**
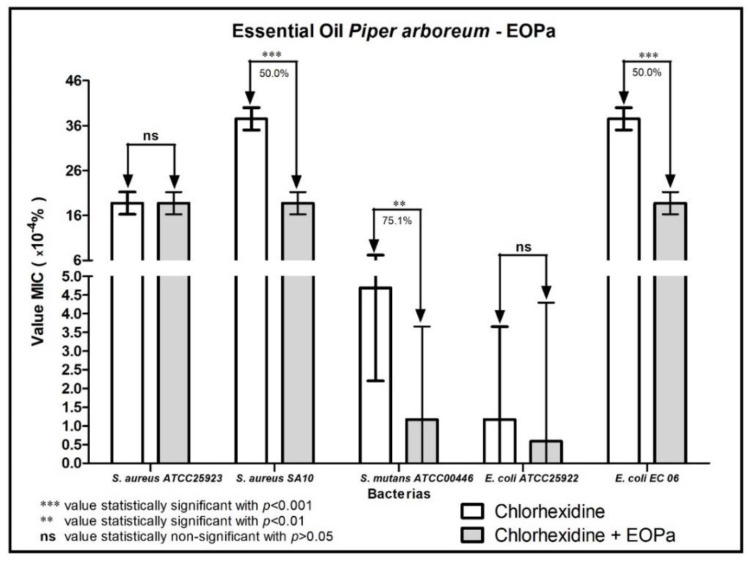
Evaluation of the modulatory activity of EOPa combined with chlorhexidine against bacterial strains.

**Figure 2:**
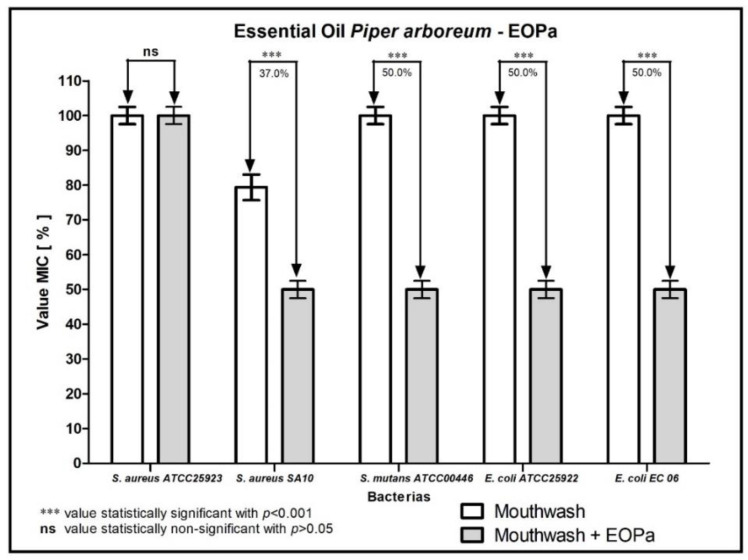
Evaluation of the modulating activity of EOPa combined with mouthwash against bacterial strains.

**Figure 3:**
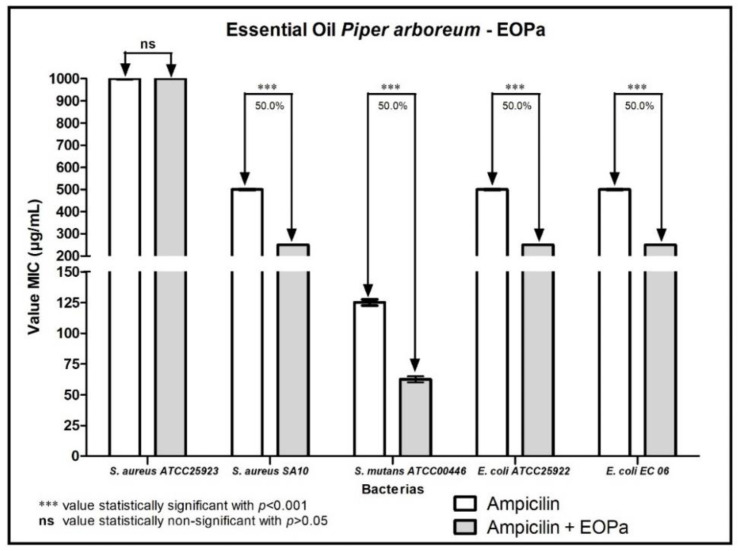
Evaluation of the modulatory activity of EOPa combined with ampicillin against bacterial strains.

**Figure 4:**
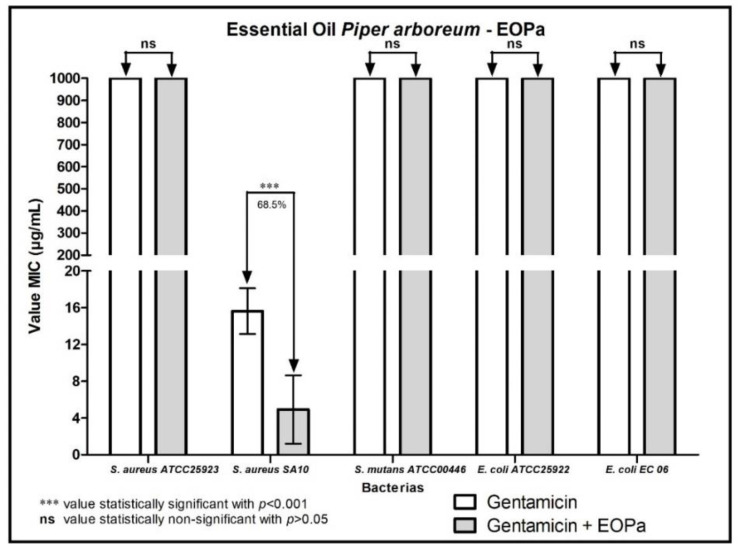
Evaluation of the modulatory activity of EOPa combined with gentamicin against bacterial strains.

**Figure 5:**
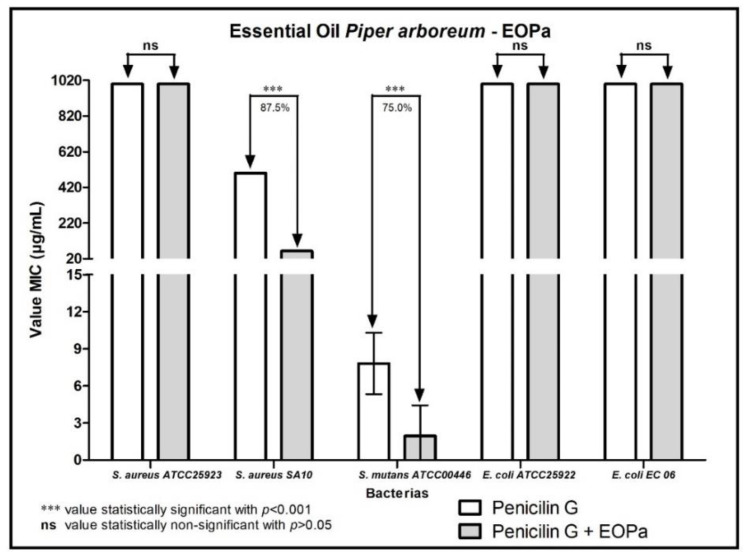
Evaluation of the modulatory activity of EOPa combined with penicillin G against bacterial strains.

The results regarding the modulating activity of bacterial resistance are organized and demonstrated in Figure 1, Figure 2, Figure 3, Figure 4 and Figure 5, thus facilitating the understanding of the combination of EOPa with chlorhexidine, mouthwash, ampicillin, gentamicin and penicillin G.

When EOPa was combined with chlorhexidine, the MIC of chlorhexidine showed a reduction of 50% against the bacterial strains *S. aureus* SA10 and *E. coli* EC06, and a reduction of the MIC of chlorhexidine by 75.1% against *S. mutans* ATCC00446, indicating a synergistic effect in the EOPa-chlorhexidine combination, as shown in Figure 1.

Figure 2 shows the result of combining EOPa with mouthwash. The mouthwash presented a 50% reduction in the MIC against the bacterial strains *S. mutans* ATCC00446, *E. coli* ATCC25922, and *E. coli* EC06, while against the bacterial strain *S. aureus* SA10, the reduction in the MIC of the mouthwash was 37%, indicating a synergistic effect of the reported combination.

Figure 3 shows the result of the combination of EOPa with ampicillin. As shown, there was a 50% reduction in the MIC of ampicillin against the bacterial strains *S. aureus* SA10, *S. mutans* ATCC00446, *E. coli* CC25922 and *E. coli* EC06, indicating a remarkable synergistic effect of EOPa combined with ampicillin.

When the combination of EOPa and gentamicin was tested, a 68.5% reduction in the MIC of gentamicin against only the bacterial strain *S. aureus* SA10 was observed, indicating a synergistic effect of the combination, as shown in Figure 4.

Figure 5 represents the results of the combination of EOPa with penicillin G. A reduction of 87.5% and 75% in the MIC of penicillin G was observed against the bacterial strains *S. aureus* SA10 and *S. mutans* ATCC00446, respectively, indicating a synergistic effect on the given combination.

## 3. Discussion

In recent years, research and investigations to identify and develop new medicines derived from natural products have intensified. Studies have reported that drugs from 225 natural sources were in the development stage, and of these, about 80% were extracted from plants [44,45]. The search for medicines and genes from nature has been promoted as a non-destructive use of habitats that promotes human health as well as supports economic development and conservation [46].

Caryophyllene oxide and the sesquiterpenes derived therefrom: spathulenol, E-caryophyllene and humulene epoxide II are found in samples of essential oils from different vegetables and have shown the ability to modulate several pharmacological activities and amplify its effect. Among these activities are antiproliferative, anti-inflammatory and antibacterial [47,48].

The substance myristicin was first identified in the seed of nutmeg (*Myristica fragrans*) and was described by French colonies in the mid-18th century on the Maluku Islands. In addition to the high concentration in this seed, myristicin can also be found in cinnamon, parsley, some types of pepper and other spices native to Asia. Nutmeg has been used since ancient times to treat anxiety, stomach cramps, nausea and diarrhea. In addition, it has been described as a food preservative, as it has antimicrobial activities as well as the following related activities: antioxidant, anti-inflammatory, analgesic, antiproliferative, insecticide and larvicide. However, when used in very high amounts, myristicin can have toxic effects, leading to liver degeneration and mental confusion, as it is toxic to the central nervous system [49].

The results of this study indicated that EOPa has no clinically relevant antibacterial activity. However, other studies have shown that although a product or natural substance does not have antibacterial potential, it can be a modulator of bacterial resistance when combined with antibiotics, thus improving its effect [50].

EOPa, when combined with antibiotics, chlorhexidine and mouthwash, separately, showed a reduction in the MIC of all products tested, indicating possible synergistic modulatory activity. In this way, the oil showed significant results when combined with other substances but did not show relevant results when tested in isolation. This may have occurred because the oil is a complex mixture of several compounds in variable amounts, allowing the oil to act on different targets [51].

Various chemical compounds (synthetic or natural) have direct antibacterial activity against many species and/or can expand the activity of an antibiotic, reverse the natural resistance of bacteria to a specific antibacterial substance, and cause inhibition of antibiotic efflux proteins across the plasma membrane and/or plasmid deletion. The potentiation of antibiotic activity or reversal of antibiotic resistance allows the classification of these compounds as antibiotic activity modifiers [52,53].

The potential modulator of antibacterial activity can be explained by a strategy known as “herbal-shotgun” or “multiple targets with multiple effects” due to the fact that natural products of plant origin have a chemically diversified constitution and can act on lipopolysaccharides (LPS) and efflux pumps to reduce vulnerability to antimicrobial drugs [54].

## 4. Materials and Methods

### 4.1. Bacterial Strains

The microorganisms used in the tests were provided by the Laboratory of Microbiology and Molecular Biology—LMBM, University Regional of Cariri—URCA, under the coordination of Prof. Dr. Henrique Douglas Melo Coutinho. Standard strains of bacteria *Staphylococcus aureus* (ATCC25923 and resistant SA10), *Escherichia coli* (ATCC25922 and resistant EC06) and *Streptococcus mutans* ATCC00446 were used.

### 4.2. Preparation and Standardization of Bacterial Inoculum

Bacteria cultures were kept at 4 °C in heart infusion agar—HIA. Before testing, the strains were transferred to the HIA medium and incubated at 35 °C for 24 h. The active bacterial strains were inoculated in brain heart infusion—BHI at the concentration recommended by the manufacturer and incubated under the same conditions mentioned above. The suspensions with bacterial growth were diluted in BHI at a concentration of 10% until 10^5^ cells/mL was obtained [55].

### 4.3. Antibiotics and Mouthwash Solutions

Chlorhexidine gluconate, gentamicin, ampicillin and penicillin G were purchased from Sigma Chemical Corporation, St. Louis, MO, USA. Commercial mouthwash (composition: water, glycerin, propylene glycol, sorbitol, tetrapotassium pyrophosphate, polysorbate 20, tetrasodium pyrophosphate, zinc citrate, PVM/MA copolymer, benzyl alcohol, sodium fluoride [225 ppm fluorine/0.05%], sodium saccharin, acid blue 3 [CI 42051]) was purchased from a drugstore. All substances were dissolved in sterile water before use.

### 4.4. Botanical Materials and Extraction of Essential Oils

Leaves from *Piper arboreum* Aubl. (P.ar.), were collected in the spring of 2019 at Bom Jesus Biological Reserve (S 25 o 13.644′/W 48 o 34.985′), municipality of Guaraqueçaba, PR, Brazil. Voucher specimens were deposited at the Herbarium of the Municipal Botanical Museum—MBM, in Curitiba, PR, Brazil, under the number 396412 (P.ar.). The plant material was collected under the authorization of the System of Authorization and Information on Biodiversity—SISBIO number 49770-2. Additionally, information about the species was registered in the National System of Management of Genetic Heritage and Associated Traditional Knowledge (SISGEN) under number A216E5A.

The leaves were dried under shadow at room temperature and submitted to hydrodistillation using a modified Clevenger apparatus [56] at the Laboratory of Chemistry and Biology of the Federal of Paraná University, Coastal Sector. The oils were separated from the hydrolates using bidistilled dichloromethane, dried with anhydrous magnesium sulfate, filtered, concentrated in a rotary evaporator, transferred to a 5 mL vial and stored in a refrigerator. The percentage of the extracting yield was determined by the ratio of oil mass and plant material mass used (*w*/*w*).

The GC-FID and GC-MS analysis of the essential oil of the *Piper arboreum* sample (EOPa) were performed using a Shimadzu 14B GC fitted with a capillary column (DB5 Supelco, 30 m × 0.25 mm i.d. × 0.25 µm film thickness) and a Perkin-Elmer Clarus 680 fitted with a capillary column (DB5 Perkin Elmer, 30 m × 0.25 mm i.d. × 0.25 µm film thickness) coupled to a Perkin-Elmer Clarus 600T, respectively.

In the GC-FID, the following analytical conditions were used: injector and detector were operating at 250 °C and 280 °C, respectively. The carrier gas was helium at a flow rate of 1 mL min^–1^, sample injection of 0.4 µL in the split mode (1:20). The oven temperature was programmed from 60 °C (0 min) to 240 °C at a 3 °C min^−1^ gradient, and held at this temperature for 2 min, resulting in a total length of analysis of 62 min. The GC-MS was performed using the following analytical conditions: sample injection (1.0 µL), carrier gas helium at a flow rate of 1 mL min^–1^, split mode (1:20), injector temperature 220 °C, ion source at 250 °C and line transfer at 240 °C. The mass selective detector was set at 70 eV and mass range of 40–400 amu. The oven temperature was programmed from 60 °C (0 min) to 246 °C at a 3 °C min^–1^ gradient, resulting in a total length of analysis of 62 min. The identification of components was made by the computer library search based on the matching of MS spectra, comparison with literature data [43] and experimental arithmetic indices (AI) [57], which were calculated using a homologous series of linear alkanes analyzed under the same GC-flame ionization detector (FID) conditions previously described. The component quantification was based on their GC peaks areas without correcting for response factors.

### 4.5. Antibacterial Tests

#### 4.5.1. Determination of Minimum Inhibitory Concentration (MIC) In Vitro by Direct Contact

The assays for the determination of the MIC of the essential oil of *Piper arboreum* (EOPa) [1024 µg/mL], antibiotics (ampicillin, gentamicin, penicillin G) [1000 µg/mL], chlorhexidine (CLX) [0.06%], and commercial mouthwash [100%] were performed using the broth microdilution method, with concentrations ranging from [C_initial_/2] to [C_initial_/11]. The bacterial suspensions were diluted 1:10 in BHI broth to obtain a final concentration of 10^5^ cells/mL [58]. Test product samples were prepared in doubled concentration, where the initial concentrations were: EOPa [1024 µg/mL], antibiotics [1000 µg/mL], chlorhexidine (CLX) [0.06%], and commercial mouthwash (EN) [100%] in relation to the initial concentration and volumes of 100 µL were serially diluted 1:1 in 10% BHI broth. In each well with 100 µL of the culture medium, a sample of bacterial suspension was diluted 1:10. Negative controls with the BHI broth, positive controls (BHI broth + inoculum) and inhibition controls using the tested products were included in the assays. The filled plates were incubated at 35 °C for 24 h [55]. To evidence the MIC of the samples, an indicator solution of sodium resazurin (Sigma) in sterile distilled water at a concentration of 0.01% (*w*/*v*) was used. After incubation, 20 µL of the indicator solution was added to each well and the plates were incubated for 1 h at room temperature. The change from blue to pink due to the reduction of resazurin indicates bacterial growth [59], helping to visualize the MIC, defined as the lowest concentration capable of inhibiting microbial growth. The presence of bacterial growth was evidenced by the unaltered blue color.

#### 4.5.2. Modulating Activity of In Vitro Antibiotic Action by Direct Contact

To evaluate the essential oil of *Piper arboreum* (EOPa) as a modulator of the antibacterial action of antibiotics (ampicillin, gentamicin, penicillin G) [1000 µg/mL], chlorhexidine (CLX) [0.06%] and commercial mouthwash (EN) 100%, the MICs were evaluated in the presence and absence of EOPa in sterile 96-well microplates.

EOPa was mixed in 10% BHI broth at sub-inhibitory concentrations (CIM/8). Antibiotic solutions were prepared with sterile distilled water in doubled concentration (1000 µg/mL) in relation to the defined initial concentration and volumes of 100 µL were serially diluted 1:1 in 10% BHI broth. In each well with 100 µL of the culture medium contains the diluted bacterial suspension (1:10). The same controls used in the MIC evaluation for the test products were used (Sato et al. 2004, modified). The filled plates will be incubated at 35 °C for 24 h and the reading was evidenced by the use of sodium resazurin as mentioned above.

#### 4.5.3. Statistical Analysis of Microbiological Tests

The MIC results obtained in triplicate in the bacterial resistance modulation tests were tabulated in a spreadsheet using Microsoft Excel 2010 software, and applying the geometric mean formula and deviation calculation, obtaining parametric data and submission to statistical analysis and significance test. 

For statistical analysis, data expressed as geometric mean ± standard error of the mean (SEM) were subjected to analysis of variance (ANOVA), followed by the Bonferroni significance test. A significant difference was considered when *p* < 0.001. The Prisma 5 for Windows Version 5.02 (GraphPad Software, San Diego, CA, USA) Software was used.

## 5. Conclusions

In view of the results presented, the chemical composition of the essential oil obtained from the leaves of *Piper arboreum* contains substances belonging to chemical classes with proven biological activity. While the antibacterial activity of EOPa did not show clinically relevant results, however when EOPa was combined with chlorhexidine, mouthwash and antibiotics (ampicillin, gentamicin and penicillin G) to assess their influence on bacterial resistance, the oil showed significant synergistic activity, reducing the MIC of the products tested from 37% to 87.5%.

Although, we recommend expanding the tests with greater variation of the EOPa concentration combinations and the products used. In addition, we recommend a toxicity assessment and in vivo tests, with the objective of developing a possible formulation of a low-cost mouthwash that is accessible to the population more vulnerable.

## Figures and Tables

**Table 1:** Chemical composition of the essential oil of *Piper arboreum* (EOPa).

No.	Constituents	RT (min) GC-MS (EOPa)	IA_exp_	IA_lit_	EOPa [%]
1	Linalool	11.34	1097	1095	0.50
2	δ-Elemene	21.19	1331	1335	1.00
3	α-Copaene	22.86	1372	1374	2.00
4	β-Elemene	23.44	1386	1389	0.93
5	E-Caryophyllene	24.65	1417	1389	5.10
6	α-Humulene	26.08	1452	1452	0.96
7	Germacrene D	27.13	1478	1484	1.20
8	β-Selinene	27.44	1486	1484	1.52
9	γ-Amorphene	27.72	1492	1495	0.60
10	α-Muurolene	27.86	1495	1495	0.50
11	α-Bulnesene	27.97	1498	1500	0.55
12	δ-Amorphene	-	1515	1509	-
13	trans-Calamenene	-	1517	1511	-
14	Myristicin	28.71	1518	1517	7.00
15	Elemol	29.81	1549	1548	2.00
16	E-Nerolidol	-	1556	1561	-
17	Spathulenol	30.88	1577	1577	6.20
18	Caryophyllene oxide	31.08	1582	1582	30.50
19	Humulene Epoxide II	32.08	1608	1608	5.10
20	1,10-Di-epi-Cubenol	32.34	1613	1618	1.87
	Identified total				67.53

IA_exp_: Experimental arithmetic retention index; IA_lit_: Literature arithmetic retention index [43]; tr: dashes (<0.1%); 1: identified only by GC-MS.

## Data Availability

Not applicable.

## Sample Availability

Samples of compounds, results and corrections have been shared and are available with all authors and confirmed for publication.
